# Metabolomics of Non-muscle Invasive Bladder Cancer: Biomarkers for Early Detection of Bladder Cancer

**DOI:** 10.3389/fonc.2018.00494

**Published:** 2018-11-02

**Authors:** Xiangming Cheng, Xiaoyan Liu, Xiang Liu, Zhengguang Guo, Haidan Sun, Mingxin Zhang, Zhigang Ji, Wei Sun

**Affiliations:** ^1^Department of Urology, Peking Union Medical College Hospital, Chinese Academy of Medical Science, Beijing, China; ^2^Core Facility of Instrument, Institute of Basic Medical Sciences, Chinese Academy of Medical Sciences/School of Basic Medicine, Peking Union Medical College, Beijing, China

**Keywords:** metabolomics, bladder cancer, non-muscle invasive, early detection, biomarker

## Abstract

**Background:** Clinical outcomes of bladder cancer (BC) are tightly associated with the stage and grade of the initial diagnosis of BC because early detection is clearly important for patients with BC. However, the diagnostic capability of current detection methods, such as urinary cytology, cystoscopy, imageology method, and several urine-based tests, is inadequate for early detection of BC. The objective of our study is to discover novel biomarkers for detecting BC at an early stage, called non-muscle invasive (NMI) BC, using liquid chromatography-high resolution mass spectrometry (LC-HRMS)-based metabolomics.

**Methods:** First, morning midstream urine samples were collected from healthy adult and NMIBC patients. The LC-HRMS-based metabolomics were applied to distinguish the NMIBC group without hematuria from the controls (gender- and age-matched volunteers with normal clinically healthy index), low-grade NMIBC from the controls, and high-grade from low-grade NMIBC.

**Results:** A total of 284 subjects were enrolled in our study including 117 healthy adults, 80 NMIBC patients without hematuria, and 87 NMIBC patients with hematuria. The metabolite panel including dopamine 4-sulfate, MG00/1846Z,9Z,12Z,15Z/00, aspartyl-histidine, and tyrosyl-methionine was found in a discovery set, which showed the predictive ability to distinguish the NMIBC group from the control group with an area under the curve (AUC) of 0.838 in an external validation set. The AUC of the panel for low-grade NMIBC samples, which consisted of 3-hydroxy-cis-5-tetradecenoylcarnitine, 6-ketoestriol, beta-cortolone, tetrahydrocorticosterone, and heptylmalonic acid, was 0.899. The sensitivity and specificity were 0.881 and 0.786, respectively. The AUC of the panel for distinction of low-grade NMIBC with and without hematuria against high-grade NMIBC with and without hematuria were 0.827 and 0.755, respectively. In addition, metabolites involved in tryptophan metabolism were upregulated in the urine of high-grade NMIBC patients when compared with low-grade NMIBC patients with the presence or absence of hematuria.

**Conclusion:** The NMIBC urine metabolic profiling was able to assist in the early detection of BC. Panels of metabolites were discovered to have a potential value for high-grade NMIBC and low-grade NMIBC diagnosis as well as for NMIBC grading distinction.

## Introduction

Bladder cancer (BC) is the ninth most common cancer and affects more than 400,000 patients annually worldwide (1). Early detection and treatment are clearly effective methods for improving the five-year survival rate, which is up to 90% for non-muscle-invasive (NMI) BC (2). BC is mostly an asymptomatic disease, particularly at its inception, which hinders its detection. Patients without hematuria or even with microscopic hematuria can have an early stage of BC (3). Due to the absence of hematuria, these individuals are not referred to a urologist; therefore, their disease is not treated at an early stage. Thus, earlier detection of the disease before the development of gross hematuria can improve the survival of patients with BC.

Current detection of BC is primarily based on urinary cytology, cystoscopy, and imageology methods [such as computed tomography (CT) and ultrasound (US)]. However, the detection of small lesions by CT and US in an incompletely filled bladder, which is a common problem in patients without hematuria, is still challenging. The diagnostic sensitivity of urinary cytology is low, only 16% for low-grade NMIBC (4, 5), and cystoscopy is invasive and costly. Currently, a number of urine-based tests, such as bladder tumor antigen (BTA), nuclear matrix protein 22 (NMP22), ImmunoCyt, and FISH (UroVysion), have been developed and approved by the Food and Drug Administration (FDA) (6). However, the diagnostic capability of these tests is insufficient, especially for NMIBC (7). Moreover, at the time of diagnosis of BC, approximately 70–80% of BC is NMIBC, while the remaining 20–30% is muscle-invasive BC (MIBC) (8). Although both cancer types originate from the urothelium in the bladder, MIBC, and NMIBC have distinct clinical characteristics and different metabolites (9). Therefore, the discovery of novel biomarkers for NMIBC screening is still an urgent necessity.

Urine is in direct contact with the bladder epithelial cells that may give rise to BC. Compared with other body fluids such as blood, urine is collected noninvasively and in large amounts. Metabolomics is a relatively new scientific field for the investigation of biochemical processes that involve metabolites. As a result, urine metabolomics has become a useful and promising strategy to identify biomarkers for BC (9–12). In 2014, high performance liquid chromatography-mass spectrometry (HPLC-MS) was used by Jin et al. to perform a urine metabolomics study in 138 BC patients against 121 controls. Within that study, 12 metabolites were successfully used to identify BC versus controls with an area under the curve (AUC) of 0.937 (9). These 12 metabolites were associated with glycolysis and beta-oxidation. Wittman et al. analyzed urine samples using ultra-HPLC-MS/MS (UHPLC-MS/MS) and gas chromatography-mass spectrometry (GC-MS) and generated a metabolite panel that was able to discern BC from noncancerous controls with an AUC of 0.81 (12). Recently, in 2017, a study of urine metabolomics compared 87 BC patients with 65 hernia controls by UPLC-time of flight-MS (UPLC-TOF-MS) and described a predictive model for BC detection based on six metabolites with an 84.76% accuracy, but only a single metabolite, imidazoleacetic acid, was identified (10).

**Figure 1:**
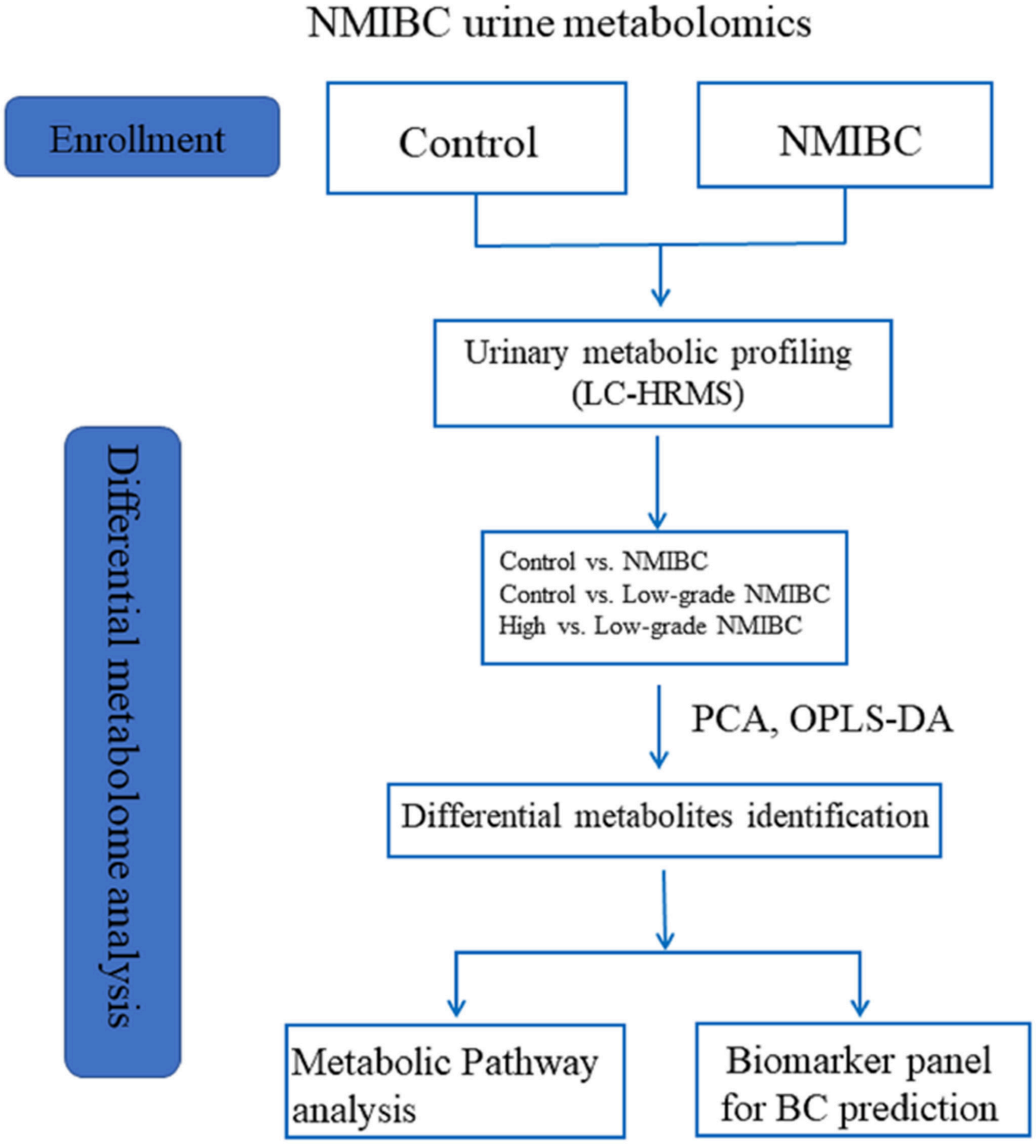
The workflow of this study.

According to our previous study (13), gender and age can influence interindividual variations in the urine metabolome. This relationship has been confirmed by other urine metabolome studies (14, 15). Furthermore, metabolites of hemoglobin in urine might also influence the diagnostic value of urine metabolomics as a biomarker for BC. Perturbations in these factors must be controlled when designing studies to obtain useful diagnostic information. However, the most recent studies have not taken these variations into consideration when discovering metabolic markers. Moreover, until now, only a few studies have reported biomarkers of NMIBC for the early detection of BC, which is more important for improving the survival rate than the detection of BC only. In the present work, liquid chromatography-high resolution mass spectrometry (LC-HRMS)-based metabolomics was applied for the early detection of BC in patients without hematuria. This study recruited the largest number of NMIBC subjects without hemoglobin in their urine. Gender- and age-matched volunteers with a normal clinically healthy index were grouped as control subjects. A combinatorial biomarker panel was defined for NMIBC diagnosis. To address the shortcomings of urine cytology for the diagnosis of low-grade NMIBC, a metabolomic approach was used as a surrogate tactic for accurate low-grade NMIBC probing. Furthermore, we were able to discover a biomarker panel that could distinguish between high-grade NMIBC with hematuria and low-grade NMIBC. The differences in metabolites found in patients with high-grade NMIBC vs. the healthy controls and in patients with high-grade NMIBC vs. patients with low-grade NMIBC may contribute to an overall understanding of the progression of NMIBC in further studies. The workflow is provided in Figure 1.

## Materials and methods

### Urine collection

This study was approved by the Institutional Review Board of the Institute of Basic Medical Sciences, Chinese Academy of Medical Sciences. Midstream urine samples were collected from healthy adult and NMIBC patients at the Peking Union Medical College Hospital, which excluded subjects with renal dysfunction and metabolic disorders. The urine samples were collected from the first urination in the morning. Samples were centrifuged within 6 h of collection; the supernatants were isolated, aliquoted, and stored at −80°C until analysis. All human subjects provided informed consent before participating in this study.

### Urine sample preparation

Urine samples were prepared using the method described in our previous study (13). In brief, 200 μl urine was mixed with 200 μl acetonitrile and vortexed for 30 s. The mixture was centrifuged at 14,000 × g for 10 min. Then, the supernatant was dried under vacuum and stored at −80°C until analysis. Before analysis, the dried powder leftover from the supernatant was reconstituted in 200 μl of 2% acetonitrile. Additionally, a small-protein depletion was performed using 10 kDa molecular weight cutoff ultracentrifugation filters (Millipore Amicon Ultra, MA) before transferring the samples to an autosampler. Quality control (QC) samples were prepared by mixing equal aliquots of 80 representative samples across the various groups undergoing the analysis. The QC samples were injected between every 10 samples throughout the analytical run to assess the stability and repeatability of the analytical process.

### LC-HRMS analysis

Urine samples were analyzed using a Waters ACQUITY H-class LC system coupled with an LTQ-Orbitrap mass spectrometer (Thermo Fisher Scientific, MA, USA). A 17 min gradient was run on a Waters HSS C18 column (3.0 × 100 mm, 1.7 μm) at a flow rate of 0.5 ml/min to separate urine metabolites. Mobile phase A was 0.1% formic acid in H_2_O and mobile phase B was acetonitrile. The gradient was set as follows: 0–1 min, 2% solvent B; 1–3 min, 2–55% solvent B; 3–8 min, 55–100% solvent B; 8–12 min, 100% solvent B; 12–12.1 min, 100–2% solvent B; and 12.1–17 min, 2% solvent B. The column temperature was 50°C. The mass scan ranged from 100 to 1,000 m/z. The MS1 analysis resolution was set as 60 K, and the MS2 resolution was 15 K. The MS1 automatic gain control target was 1 × 10^6^, and the maximum injection time (IT) was 100 ms. The MS2 automatic gain control target was set as 5 × 10^5^ and the maximum IT was 50 ms. Higher-energy collisional dissociation (HCD) fragmentation mode was used to dissociate differential metabolites with the optimal collision energy of 20, 40, 60, or 80.

### Normalization of data

Urine MS data were normalized using the “normalize to all compounds” method to eliminate the bias of the sampling and analysis system. The normalization method includes the following steps:

1. Normalization reference (the “target”): one run is automatically selected as the normalization reference.
2. Log10 ratio calculation: for every run, a ratio of the value of the compound ion abundance in that run to the value in the normalization reference was calculated. This ratio calculation removes the influence of absolute abundance from the process and has a major advantage over total-abundance-based methods.
3. Scalar estimation in log space: the next step is to center the log10 ratio distributions onto that of the normalization reference for each sample run.
4. Scalar application: once the scalar has been derived in the log space and returned to an “abundance-space ratio,” the value can be used to normalize all values in the sample run; this completes the process.

### Data processing

Mass Spectrometry raw data were processed by Progenesis QI (Waters, Milford, MA, USA) software (16, 17). The detailed workflow is given in the Supplementary Material. The exported feature file was imported to MetaboAnalyst 3.0 (http://www.metaboanalyst.ca) to perform missing value estimation, log transformation, and Pareto scaling. Variables missed in 50% or more samples were removed from further statistical analysis. The Wilcoxon rank-sum test was used to evaluate the significance of the variables. False discovery rate (FDR) correction was used to estimate the chance of false positives and correct values for multiple hypothesis testing. The cutoff was set as 0.05. Principal component analysis (PCA) and orthogonal partial least squares discriminant analysis (OPLS-DA) were performed using SIMCA 14.0 (Umetrics, Sweden) software. Differential variables were selected according to the following: (1) adjusted *P*-value < 0.05; (2) fold change > 1.5; and (3) VIP value >1.0. The receiver operator characteristic curve (ROC) analysis was used to evaluate the prediction accuracy of metabolites for BC.

## Results

### Subjects

A total of 284 subjects were enrolled in our study including 117 healthy adults as the control group, 80 NMIBC patients without hematuria, and 87 NMIBC patients with hematuria. The detailed clinical information for the patients is listed in Table S1. All the NMIBC cases were histopathologically proved to have transitional cell carcinoma without any other histologic variants such as squamous cell carcinoma, adenocarcinoma, metaplasia, etc. For early detection, biomarkers for NMIBC were discovered based on differential analysis of 54 age- and gender-matched patients and 78 control samples and then were validated in another batch of samples consisting of 26 NMIBC samples and 39 control samples. In an attempt to discover potential biomarkers of low-grade NMIBC, 43 low-grade NMIBC samples and 43 age- and gender-matched control samples were differentially analyzed. Furthermore, urinary metabolic profiling differences between the low-grade and high-grade NMIBC samples were also analyzed. For this study, 43 samples of low-grade NMIBC and 37 samples of high-grade NMIBC without hematuria were used, as well as 18 of low-grade and 69 of high-grade NMIBC with hematuria. The details of the subjects are shown in Table 1.

**Table 1:** Clinical information for the subjects enrolled in exploration for early detection of NMIBC.

	**NMIBC vs. control**				**Low-grade NMIBC vs. control**		**Low-grade NMIBC vs. High-grade NMIBC**	
	**Discovery set**		**External validation set**		**Low-grade NMIBC**	**Control**	**Low-grade NMIBC**	**High-grade NMIBC**
	**NMIBC**	**Control**	**NMIBC**	**Control**			**With/without hematuria**	**With/without hematuria**
Cases	54	78	26	39	43	43	18/43	69/37
Age	62.2 ± 13.2	59.5 ± 11.2	64.0 ± 11.3	59.7 ± 11.3	59.7 ± 13.5	59.8 ± 12.8	65.4 ± 10.7/59.7 ± 13.5	68.9 ± 10.8/66.5 ± 10.4
Sex (male/female)	42/12	61/17	21/5	30/9	33/10	33/10	(12/6)/(33/10)	(55/14)/(30/7)
Grade (low/high)	29/25		14/12		42/0		(18/0)/(43/0)	(0/69)/(0/37)

### Quality control

All samples were analyzed randomly over approximately 5 days. The stability of the system was assessed by the QC sample, which is an important quality-control process in large-scale metabolomic studies. Overall, 24 QC injections were analyzed. Variations in the QC sample with time were plotted to evaluate the technical reproducibility. The results showed a stable condition with small variation (< ± 2SD) (Figure S1A). In addition, tight clustering of the QC samples (Figure S1B) demonstrated good consistency in the QC data. The results suggested that group differences result from biological variations rather than from an analysis bias.

### NMIBC biomarker discovery

To define whether hematuria influences the components of the urinary metabolome and the analysis of various BC groups, PCA analysis was performed in the urine of the BC patients with or without hematuria. As shown in Figure S2, there was a significant difference between metabolites in subjects with and without hematuria. Because the control samples were without hematuria, the subjects without hematuria were used for NMIBC and low-grade NMIBC biomarker discovery. Differences between the low- and high-grade NMIBC samples were explored in two groups of samples divided based on the presence or absence of hematuria.

### Analysis of NMIBC vs. the control group

**Figure 2:**
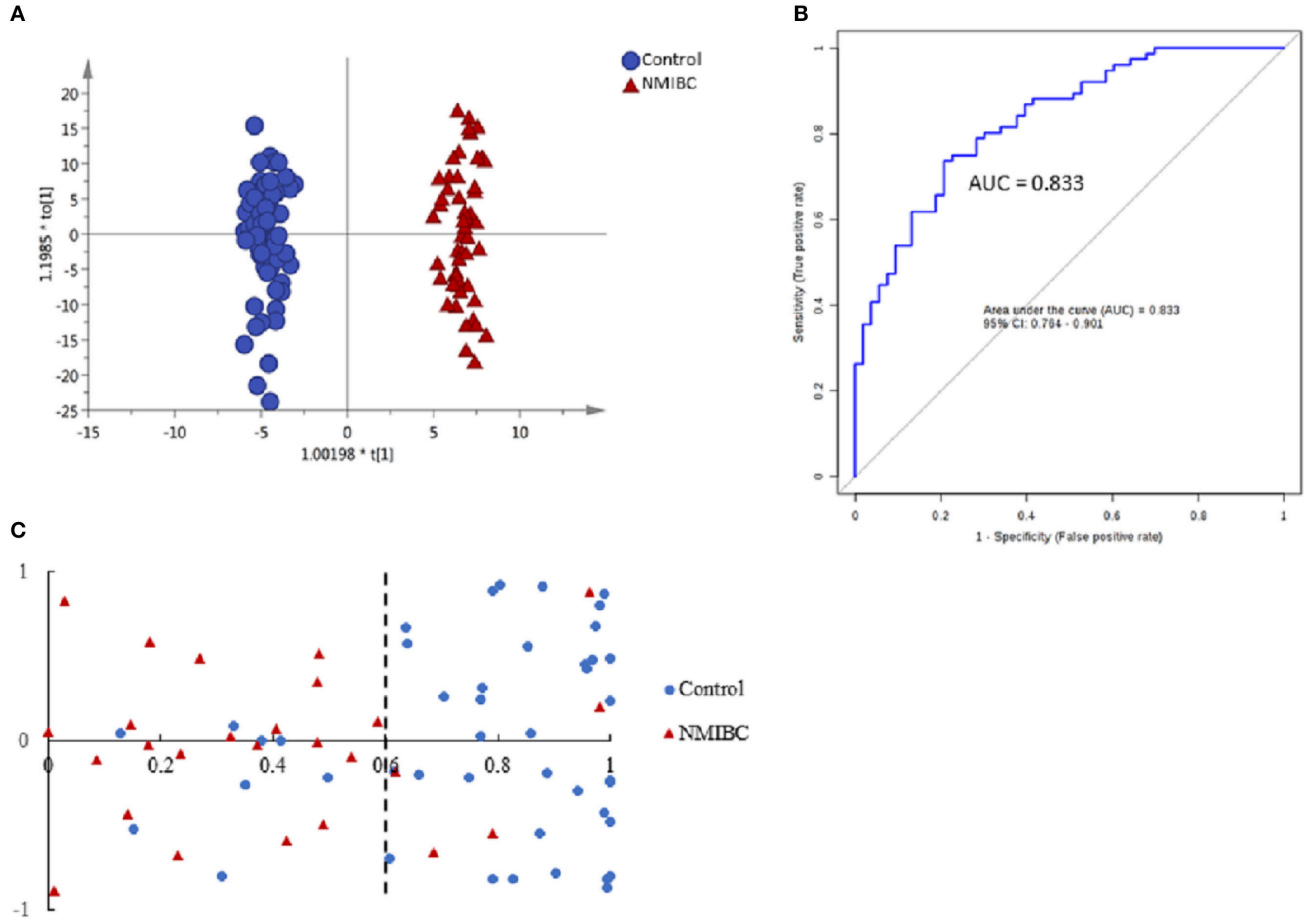
Analysis of metabolic profiling in NMIBC as compared with control group. **(A)** Score plot of OPLS-DA model based on metabolome between NMIBC and control group. **(B)** ROC plot based on model to quantify the discrimination degree of NMIBC and control group. **(C)** External prediction accuracy of NMIBC prediction model established by a metabolite panel of Dopamine 4-sulfate, MG00/1846Z,9Z,12Z,15Z/00, Aspartyl-Histidine, Tyrosyl-Methionine.

First, unsupervised PCA analysis was performed to visualize metabolic profiling differences between the control and NMIBC subjects (Figure S3A). The score plot suggested an apparent discrimination between the two groups with AUC > 0.8. Then, a supervised OPLS-DA model was established (Figure 2A) and differential metabolites were selected according to the value importance plot (VIP) value (VIP > 1). Finally, 21 significantly differential metabolites were identified (Table S2). The data indicate that metabolites involved in fatty acid metabolism were upregulated in NMIBC patients, whereas metabolites involved in the amino acid degradation pathway, carbofuran metabolism, and fatty acid oxidation were downregulated.

The ROC curves were used to evaluate the diagnostic accuracy of the differential metabolites for NMIBC. The results showed that 15 metabolites have potential clinical diagnostic value with AUC values above 0.7, and 2 metabolites have good diagnostic value with AUC values above 0.8 (Table S3). Metabolite panels are known to have more predictive power than single metabolites (18). Multivariate ROC curve-based exploratory analysis was used (http://www.metaboanalyst.ca/faces/upload/RocUploadView.xhtml). A metabolite panel consisting of dopamine 4-sulfate, MG00/1846Z,9Z,12Z,15Z/00, aspartyl-histidine, and tyrosyl-methionine was found to have the best prediction accuracy for NMIBC. The AUC was 0.857 for the testing dataset and 0.833 for 10-fold cross-validation (Figure 2B). Then, an external validation was performed to validate the model. The AUC was 0.838. The sensitivity and specificity were 0.807 and 0.818, respectively (Table 2). The model could predict correctly 21 out of 26 NMIBC patients and 31 out of 39 control group subjects (Figure 2C) with an accuracy of 80% for the prediction of NMIBC without hematuria.

**Table 2:** Performance of Logistic Regression Model for NMIBC discrimination.

	**AUC**	**Sensitivity**	**Specificity**
^a^**NMIBC vs. CONTROL**			
Training/discovery	0.857 (0.837~0.878)	0.754 (0.722~0.787)	0.786 (0.749~0.823)
10-fold cross-validation	0.833 (0.764~0.901)	0.737 (0.737~0.836)	0.792 (0.683~0.902)
External validation	0.838 (0.769~0.953)	0.807 (0.730~0.853)	0.818 (0.769~0.940)
^b^**LOW-GRADE NMIBC vs. CONTROL**			
Training/discovery	0.938 (0.923~0.953)	0.899 (0.869~0.930)	0.791 (0.750~0.832)
10-fold cross-validation	0.899 (0.836~0.963)	0.881 (0.881~0.979)	0.786 (0.662~0.910)
^c^**HIGH- AND LOW-GRADE NMIBC WITHOUT HEMATURIA**			
Training/discovery	0.802 (0.770~0.834)	0.751 (0.708~0.795)	0.757 (0.711~0.803)
10-fold cross-validation	0.755 (0.645~0.866)	0.762 (0.762~0.891)	0.757 (0.619~0.895)
^d^**HIGH- and LOW-GRADE NMIBC WITH HEMATURIA**			
Training/discovery	0.878 (0.852~0.904)	0.858 (0.804~0.912)	0.668 (0.631~0.705)
10-fold cross-validation	0.827 (0.731~0.923)	0.889 (0.889~1.000)	0.667 (0.555~0.778)

a *The panel: Dopamine 4-sulfate, MG00/1846Z,9Z,12Z,15Z/00, Aspartyl-Histidine, Tyrosyl-Methionine*.

b *The panel: 3-Hydroxy-cis-5-tetradecenoylcarnitine, 6-Ketoestriol, Beta-Cortolone, Tetrahydrocorticosterone, Heptylmalonic acid*.

c *The panel: N-Acetyl-4-O-acetylneuraminic acid, 4-(2-Aminophenyl)-2,4-dioxobutanoic acid, 6-Keto-decanoylcarnitine, 3-hydroxydecanoyl carnitine, 2-Hydroxylauroylcarnitine*.

d *The panel: Indolylacryloylglycine, Histidinyl-Histidine, Indoleacrylic acid, N-acetyl-5-methoxykynuramine, L-3-Hydroxykynurenine*.

### Analysis of low-grade NMIBC vs. the control group

**Figure 3:**
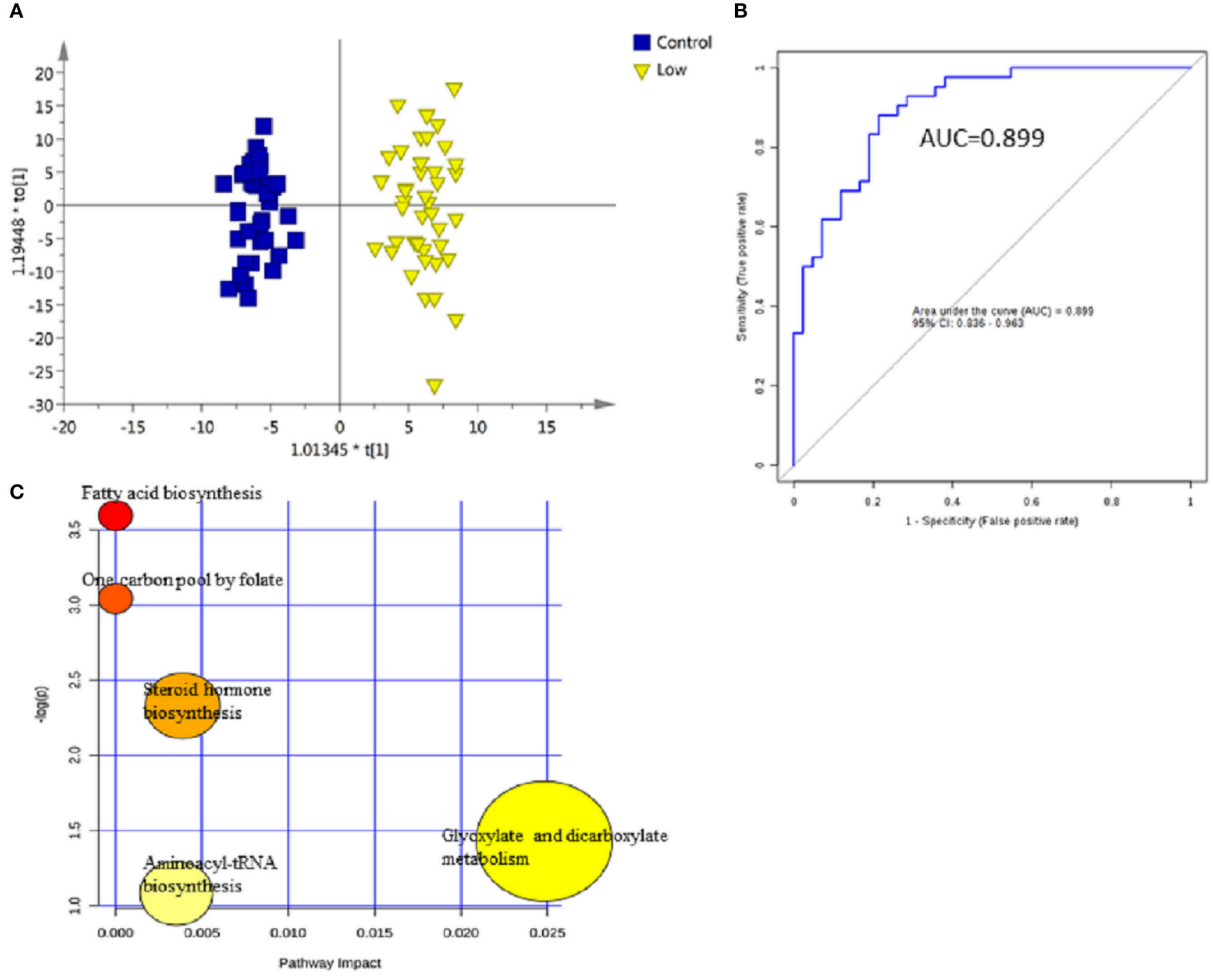
Analysis of metabolic profiling in low-grade NMIBC compared with control group. **(A)** Score plot of OPLS-DA model based on metabolome between low-grade NMIBC and control group. **(B)** ROC plot with 10-fold cross-validation based on model of 3-Hydroxy-cis-5-tetradecenoylcarnitine, 6-Ketoestriol, Beta-Cortolone, Tetrahydrocorticosterone, and Heptylmalonic acid to quantify the discrimination degree of low-grade NMIBC and control group. **(C)** Pathway analysis of differential metabolites.

For early detection, additional differences between the low-grade NMIBC group and the healthy group had to be explored. A PCA was performed, and the analysis showed apparent discrimination between the control samples and the low-grade NMIBC samples with AUC values above 0.8 (Figure S3B). Furthermore, an OPLS-DA model was established for differential metabolite selection (Figure 3A). As a result, a total of 51 significantly differential metabolites were identified (Table S4). The data showed that metabolites involved in purine biosynthesis and fatty acid metabolism were upregulated in the low-grade NMIBC patients, whereas metabolites involved in steroid hormone biosynthesis and protein digestion were downregulated. Pathway power analysis indicated that fatty acid biosynthesis, steroid hormone biosynthesis, glyoxylate and dicarboxylate metabolism, steroid hormone biosynthesis, and aminoacyl-tRNA biosynthesis significantly contributed to the low-grade NMIBC distinction (Figure 3C). A total of 47 metabolites had good diagnostic value, with AUC above 0.7 and 7 metabolites had their AUC values above 0.8 (Table S5). A metabolite panel consisting of 3-hydroxy-cis-5-tetradecenoylcarnitine, 6-ketoestriol, beta-Cortolone, tetrahydrocorticosterone, and heptylmalonic acid was used to construct a robust model for distinction between the healthy group and the low-grade NMIBC group. The AUC of the panel was 0.938 for the testing dataset and 0.899 for 10-fold cross-validation (Figure 3B). The sensitivity and specificity were 0.881 and 0.786, respectively (Table 2).

### Difference of urine metabolomics between the high- and low-grade NMIBC

#### Samples without hematuria

**Figure 4:**
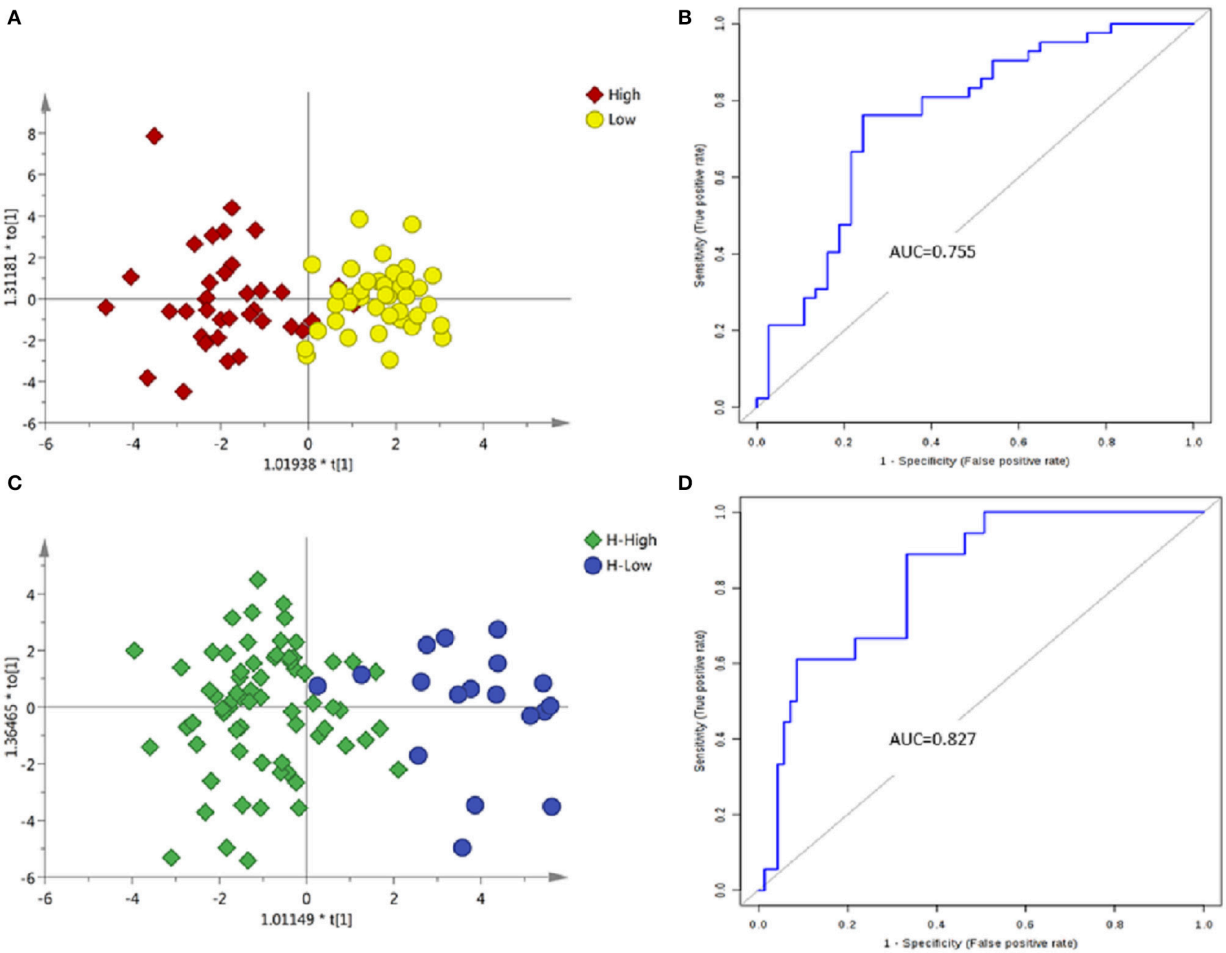
Analysis of metabolic profiling in low- and high-grade NMIBC. **(A)** Score plot of OPLS-DA model based on metabolome between low- and high-grade NMIBC without hematuria. **(B)** ROC plot based on model to quantify the discrimination degree of NMIBC grading without hematuria. **(C)** Score plot of OPLS-DA model based on metabolome between low- and high-grade NMIBC with hematuria. **(D)** ROC plot based on model to quantify the discrimination degree of NMIBC grading with hematuria.

The PCA between the high-grade and low-grade NMIBC samples (Figure S3C) did not suggest apparent separation. An OPLS-DA model suggested apparent discrimination between the two groups (Figure 4A). Differential metabolites were selected based on the VIP value of OPLS-DA. Finally, 15 significantly differential metabolites were identified (Tables S6, S7). The data showed that metabolites involved in tryptophan metabolism were upregulated in the high-grade NMIBC patients when compared with the metabolites for the low-grade NMIBC patients. Using a logistic regression algorithm, a metabolite panel consisting of N-acetyl-4-O-acetylneuraminic acid, 4-(2-aminophenyl)-2,4-dioxobutanoic acid, 6-keto-decanoylcarnitine, 3-hydroxydecanoyl carnitine, and 2-hydroxylauroylcarnitine was established. The AUC of this metabolite panel was 0.802 for the testing dataset and 0.755 for 10-fold cross-validation (Figure 4B). The sensitivity and specificity were 0.762 and 0.757, respectively (Table 2).

#### Samples with hematuria

Using the same strategy as above, unsupervised PCA was performed to visualize the metabolomic differences between the high-grade and low-grade NMIBC samples with hematuria (Figure S3D), and discrimination could be observed with an AUC of approximately 0.8. Then, an OPLS-DA model was used for differential metabolites selection (Figure 4C). Ten significantly differential metabolites were identified (Tables S8, S9). The data showed that metabolites involved in tryptophan metabolism were upregulated in the high-grade NMIBC patients when compared with the metabolites for the low-grade patients. A metabolite panel consisting of indolylacryloylglycine, histidinyl-histidine, indoleacrylic acid, N-acetyl-5-methoxykynuramine, and L-3-hydroxykynurenine was found to have good distinction between the high- vs. low-grade NMIBC. The AUC was 0.878 for the testing dataset and 0.827 for 10-fold cross-validation (Figure 4D). The sensitivity and specificity were 0.889 and 0.667, respectively (Table 2).

## Discussion

The rapid development of high-throughput chemical analysis techniques such as MS has promoted urine metabolomics as a promising approach for the early detection of BC. However, few studies have excluded the influence of hemoglobin metabolites in urine as well as in age and gender. Furthermore, early noninvasive detection of BC is still a challenge. In the present study, subjects with NMIBC were classified depending on the presence or absence of hematuria to discover a metabolite panel for differentiation from the gender- and age-matched control group.

### Analysis of NMIBC vs. the control group

The results of our study indicated that metabolites involved in fatty acid metabolism were increased in the urine from patients with NMIBC when compared with that of the control group, whereas metabolites involved in fatty acid oxidation were decreased in agreement with the Warburg hypothesis (19, 20), proposing aerobic glycolysis as an important factor in tumor growth. The hypothesis considers the facilitation of tumor initiation by dysregulation of lipid metabolism and the increased utilization of glucose for energy as the hallmarks of many proliferating tumors. Moreover, a mutation of the mitochondrial cytochrome B gene, thus interrupting the oxidative phosphorylation system, has been reported in BC (21).

Dopamine 4-sulfate, a metabolite of endogenous dopamine, was one of the metabolites in the panel. It has been reported that dopamine influences the behavior of tumor, including ovarian carcinoma, gastric cancer, breast cancer, and colon cancer (22), by inhibiting cell proliferation. Thus, the metabolism of dopamine can be increased in cancer, and this has been reported in neuroblastoma and Wilms' tumors (23). Our study is the first to report high levels of metabolism of dopamine in NMIBC apart from bladder pheochromocytoma. MG00/1846Z,9Z,12Z,15Z/00, another metabolite in the panel, is a monoacylglyceride that can be broken down by monoacylglycerol lipase. In the present study, the level of monoacylglyceride was low probably due to the high level of monoacylglycerol lipase, which has been shown to promote hepatocellular carcinoma and colorectal cancer (24, 25).

Defective xenobiotic metabolism has been reported in BC patients and has mainly been attributed to the defects in the genes related to regulation of detoxification processes (26). The present study demonstrated a decrease in carbofuran metabolism in NMIBC patients, thus further validating the defective metabolism of xenobiotics in NMIBC. We measured lower levels of avocadyne 4-acetate and 1-acetoxy-2-hydroxy-16-heptadecen-4-one in the urine from patients with NMIBC than in the urine from healthy controls. Both of these metabolites are members of the class of compounds known as long-chain fatty alcohols, which are present in fruits, supporting the impact of dietary habits on BC (27, 28).

### Analysis of low-grade NMIBC vs. the control group

A panel of five urinary metabolites was discovered with high sensitivities at or above 85% that did not compromise the specificities for distinguishing low-grade NMIBC from the control group. The significantly differential metabolites included undecanoylcarnitine, 3-hydroxytetradecanoyl carnitine, 3-hydroxy-5, 8-tetradecadiencarnitine, 3-hydroxy-cis-5-tetradecenoylcarnitine, and O-decanoyl-L-carnitine; the levels of these compounds were decreased in the low-grade NMIBC group without the elevation of any other carnitines. Since carnitine and its short-chain derivatives are essential for the entry of fatty acid into mitochondria for oxidation, fatty acid oxidation is decreased in the low-grade NMIBC group compared to the controls. Moreover, acetyl-carnitine provides a source of acetyl groups for nuclear protein acetylation by histone acetyl-transferases (29). It was reported that acetylation of the transcription factor p53 by histone acetyl transferase p300/CBP is required for p53 activation (30, 31). Additionally, Pisano et al. also demonstrated that acetyl-carnitine had a direct anti-tumor effect through the potentiation of platinum-based therapy (32). In our study, the level of undecanoylcarnitine, a member of acetyl-carnitine family, was decreased in the low-grade NMIBC group compared to the control group.

Myristic acid, an exogenous saturated fatty acid that originates from dietary uptake and is involved in the biological process of energy production (33, 34), was increased in the urine of the low-grade NMIBC patients, further illustrating the lowered level of fatty acid oxidation in the low-grade NMIBC samples vs. the controls. Moreover, exogenous myristic acid can increase the biosynthesis of myristoyl CoA and myristoylated Src and promote Src kinase-mediated oncogenic signaling in the human cells (35).

#### Difference in urine metabolomics between high- and low-grade NMIBC

So far, there is no urine-based metabolomics approach to differentiate between high-grade and low-grade NMIBC, although high-grade NMIBC is obviously more aggressive than low-grade NMIBC. In the current study, we attempted to find a difference in the metabolome between low-grade NMIBC and high-grade NMIBC. High levels of L-3-hydroxykynurenine and 5-hydroxyindoleacetaldehyde were observed in high-grade NMIBC with hematuria; both metabolites are associated with tryptophan metabolism. Furthermore, L-3-hydroxykynurenine is a metabolite in the kynurenine pathway, the major route of tryptophan metabolism. Increased concentrations of kynureninic acid have been detected in many cancer types, including colon carcinoma, non-small cell lung cancer, etc (36–38). Interestingly, kynureninic acid was increased in patients with metastases that spread to lymph nodes when compared with nonmetastatic patients (37). Moreover, a tryptophan catabolic enzyme, indoleamine 2,3-dioxygenase, has been reported as a central driver of malignant development and progression (39). Our metabolomic analysis of NMIBC grades revealed an association between tryptophan metabolism and the progression of BC. We also observed a decrease in 6-keto-decanoylcarnitine, a member of the acyl carnitine family, in high-grade NMIBC without hematuria vs. low-grade NMIBC. A similar reduction in acyl carnitine metabolites was found in low-grade NMIBC samples compared to the control group.

According to our study, the prediction accuracy of the metabolite panel for high-grade NMIBC vs. low-grade NMIBC was lower for NMIBC without hematuria than for NMIBC with hematuria. The following reasons may explain this observation: (1) the subjects in two groups without hematuria were not age-matched; (2) NMIBC without hematuria may occur in the earlier period of BC initiation, so the difference may not yet be obvious in the metabolome.

## Conclusion

Thus, a comprehensive characterization of NMIBC urine metabolome was conducted in our study. A pilot study of NMIBC urine metabolomic profiling was able to distinguish the NMIBC group from the controls, low-grade NMIBC from the controls, and high-grade NMIBC from low-grade NMIBC. Panels of metabolites were discovered to have potential value for high-grade NMIBC and low-grade NMIBC diagnosis as well as for NMIBC grading distinction. Our data will not only benefit the application of the urine metabolome in disease biomarker discovery but also contribute to the exploration of the initiation of NMIBC. Further studies, including a blood metabolomics study and a multicenter metabolomic study of BC, are required to generate and validate a robust method for the detection of early BC.

## Ethics approval and consent to participate

This study was carried out in accordance with the recommendations of the committee of Peking Union Medical College Hospital with written informed consent from all subjects. All subjects gave written informed consent in accordance with the Declaration of Helsinki. The protocol was approved by the committee of Peking Union Medical College Hospital.

## Author contributions

XC performed experiments, analyzed data, and wrote the paper. ZJ and WS initiated and organized the study. XianL and XiaoL designed the experiments. HS critically revised the manuscript. MZ and ZG analyzed the data. All authors read and approved the final version of the manuscript.

### Conflict of interest statement

The authors declare that the research was conducted in the absence of any commercial or financial relationships that could be construed as a potential conflict of interest.

## Abbreviations

BC: bladder cancer
NMIBC: non-muscle invasive bladder cancer
AUC: area under the curve
LC-HRMS: liquid chromatography- high resolution mass spectrometry
QC: quality control
PCA: principal component analysis
OPLS-DA: orthogonal partial least squares discriminant analysis partial least squares discriminate analysis
ROC: receiver operator characteristic curve
VIP: variable importance plots
CV: coefficient of variation.
