# Supplementary material for: Metabolomics of Non-muscle Invasive Bladder Cancer: Biomarkers for Early Detection of Bladder Cancer
Source: Front Oncol. 2018 Nov 2;8:494. doi: 10.3389/fonc.2018.00494 (PMC6224486; doi:10.3389/fonc.2018.00494)
Supplement: Supplementary file 1 [file Table_1.docx]

**Supplementary materials**

**Fig. S1** Assessment of QC samples. **a.** Trend plot showing the variation of t [1] over all QC Samples. X axis numbers represented sample number , Y axis was arbitrary (3 s.d.); **b.** PC1 versus PC2 of test samples and QC samples.

**Fig. S2** PCA score plot of metabolic profiling variation between NMIBC with and without hematuria. a. Analysis of NMIBC between subjects with and without hematuria. b. Analysis of high-grade NMIBC. C. Analysis of low-grade NMIBC

**Fig. S3** PCA score plot of metabolic profiling variation. a. Analysis of NMIBC compared with control group. b. Analysis of low-grade NMIBC compared with control group. c. Analysis of difference between high- and low-grade NMIBC without hematuria. d. Analysis of difference between high- and low-grade NMIBC with hematuria

**Table S1** Clinical information of NMIBC

**Table S2** Differential metabolites between NMIBC and control group

**Table S3** Differential metabolites for NMIBC and control group distinction

**Table S4** Differential metabolites between low-grade NMIBC and control group

**Table S5** Differential metabolites for low grade NMIBC distinction

**Table S6** Differential metabolites between high and low grade NMIBC without hematuria

**Table S7** Differential metabolites for high and low grade NMIBC without hematuria distinction

**Table S8** Differential metabolites between high and low grade NMIBC with hematuria

**Table S9** Differential metabolites for high and low grade NMIBC with hematuria distinction

**Fig S1**

**
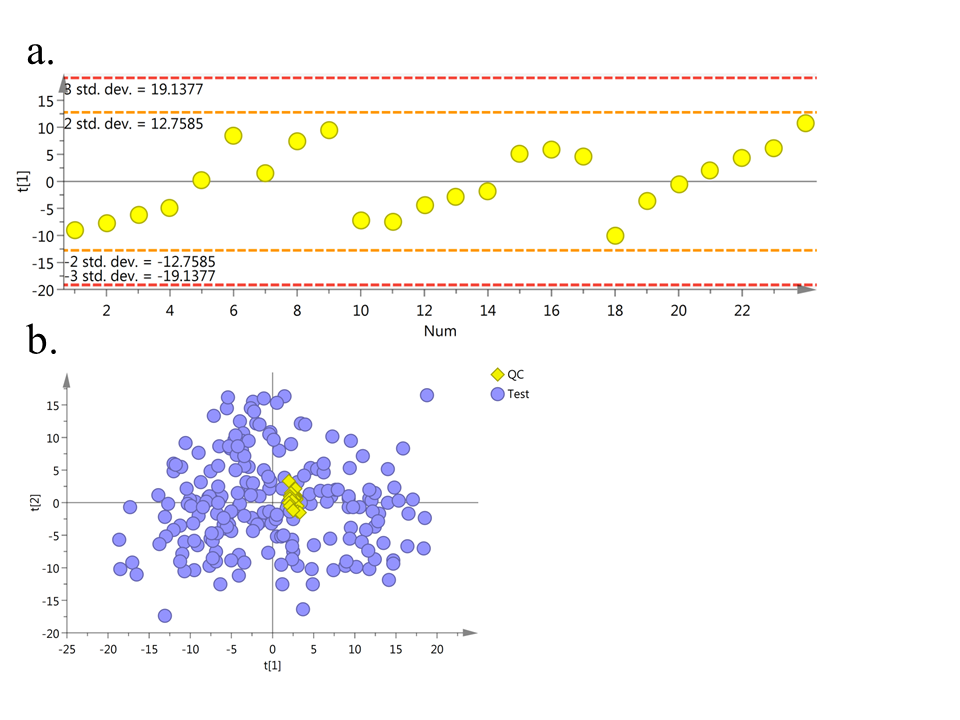
**

**Fig S2**

**
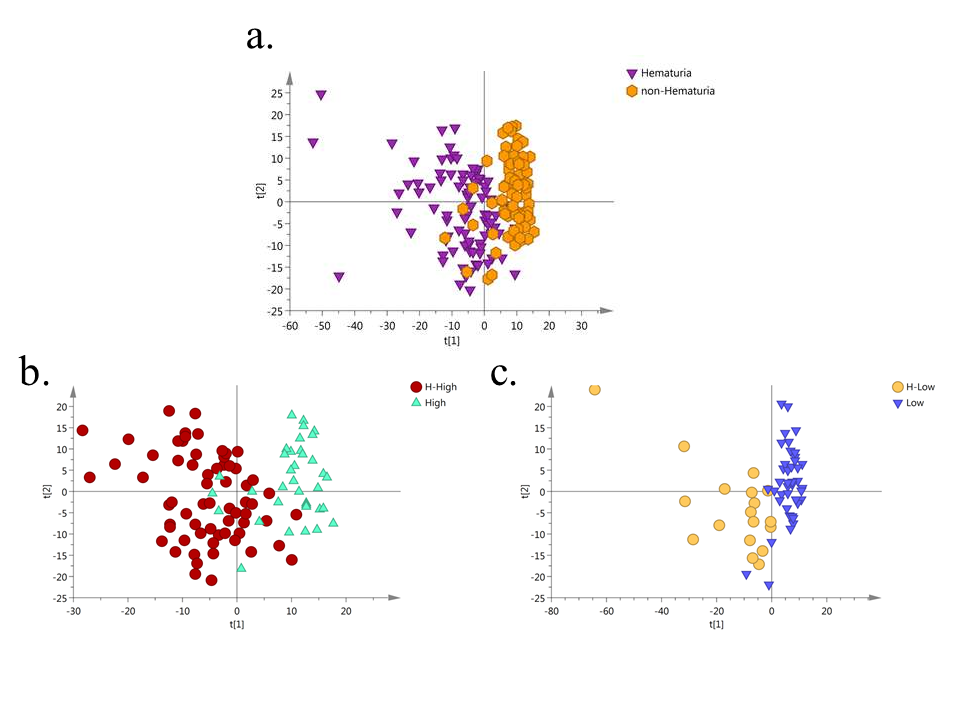
**

**Fig S3**

**
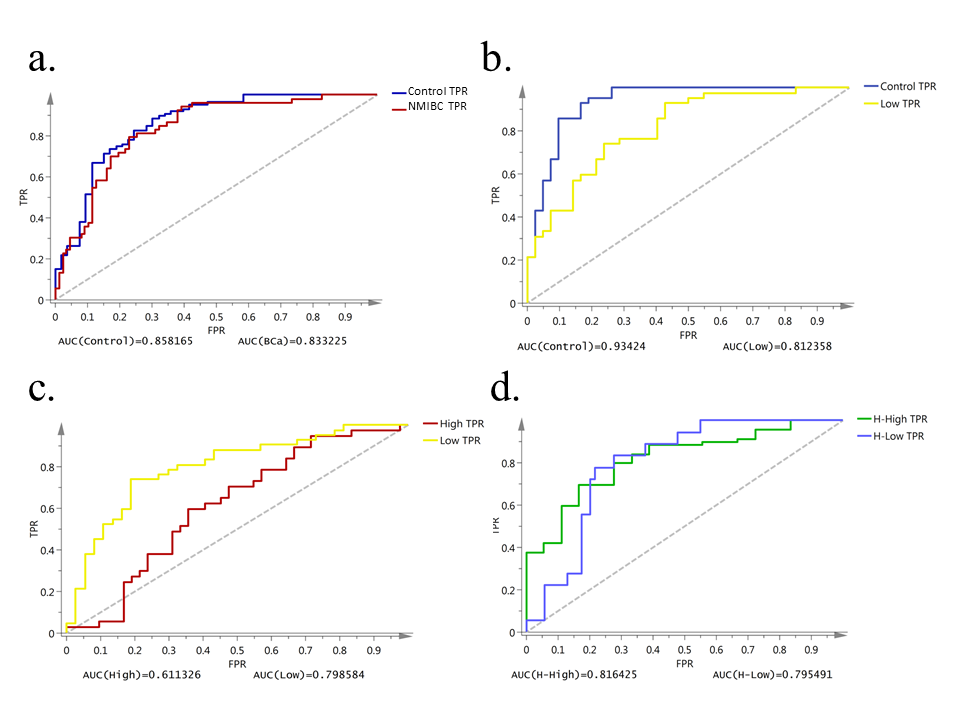
**

**Table S1** Clinical information of NMIBC: a. NMIBC without hematuria; b. NMIBC with hematuria

1. NMIBC without hematuria

| Num | Sex | Age | Weight（Kg） | Height（cm） | Grade | Red cell（cell/μL） |
| --- | --- | --- | --- | --- | --- | --- |
| 10 | M | 70 | 64 | 168 | Low | 0 |
| 29 | F | 51 | 56 | 158 | Low | 0 |
| 32 | M | 63 | 86 | 169 | Low | 0 |
| 39 | F | 79 | 79 | 155 | Low | 0 |
| 41 | M | 27 | 91 | 171 | Low | 0 |
| 44 | M | 61 | 92 | 160 | Low | 0 |
| 48 | F | 68 | 68 | 157 | Low | 0 |
| 58 | M | 40 | 83 | 178 | Low | 0 |
| 61 | F | 86 | 75 | 158 | Low | 0 |
| 76 | M | 62 | 96 | 171 | Low | 0 |
| 77 | F | 68 | 55 | 155 | Low | 0 |
| 82 | M | 84 | 67 | 170 | Low | 0 |
| 87 | F | 62 | 56 | 155 | Low | 0 |
| 96 | F | 37 | 63.5 | 157 | Low | 0 |
| 106 | M | 46 | 83 | 178 | Low | 0 |
| 116 | M | 64 | 81 | 168 | Low | 0 |
| 117 | M | 53 | 60 | 173 | Low | 0 |
| 125 | M | 61 | 64 | 170 | Low | 0 |
| 126 | F | 61 | 73 | 150 | Low | 0 |
| B1 | M | 64 | 52 | 165 | Low | 0 |
| B101 | M | 56 | 88 | 175 | Low | 0 |
| B103 | M | 32 | 72 | 170 | Low | 0 |
| B107 | M | 53 | 75 | 172 | Low | 0 |
| B121 | F | 82 | 60 | 155 | Low | 0 |
| B124 | M | 61 | 58 | 171 | Low | 0 |
| B136 | M | 81 | 71 | 170 | Low | 0 |
| B28 | M | 54 | 75 | 176 | Low | 0 |
| B35 | M | 60 | 73 | 175 | Low | 0 |
| B4 | M | 72 | 66 | 166 | Low | 0 |
| B79 | M | 51 | 68 | 165 | Low | 0 |
| B91 | M | 49 | 74 | 173 | Low | 0 |
| BCaL | M | 63 | 65 | 172 | Low | 0 |
| 107 | F | 44 | 50 | 158 | Low | 0 |
| 124 | M | 61 | 77 | 170 | Low | 0 |
| 140 | M | 72 | 72 | 166 | Low | 0 |
| B43 | M | 37 | 88 | 175 | Low | 0 |
| B9 | M | 67 | 65 | 171 | Low | 0 |
| B141 | M | 54 | 79 | 68 | Low | 0 |
| B143 | M | 59 | 76 | 176 | Low | 0 |
| B173 | M | 66 | 94 | 173 | Low | 0 |
| B177 | M | 54 | 76 | 170 | Low | 0 |
| B165 | M | 58 | 52.5 | 168 | Low | 0 |
| B81 | M | 72 | 74 | 173 | Low | 0 |
| 5 | F | 70 | 85 | 165 | High | 0 |
| 18 | M | 68 | 68 | 176 | High | 0 |
| 21 | F | 64 | 58 | 150 | High | 0 |
| 33 | M | 64 | 81 | 160 | High | 0 |
| 49 | M | 68 | 63 | 166 | High | 0 |
| 57 | M | 58 | 63 | 173 | High | 0 |
| 59 | M | 58 | 87 | 165 | High | 0 |
| 69 | F | 63 | 73 | 166 | High | 0 |
| 86 | M | 66 | 71 | 160 | High | 0 |
| 94 | M | 52 | 85 | 184 | High | 0 |
| 113 | M | 65 | 78 | 175 | High | 0 |
| 120 | M | 75 | 62 | 165 | High | 0 |
| 129 | M | 52 | 68 | 170 | High | 0 |
| 137 | M | 53 | 70 | 172 | High | 0 |
| 142 | M | 85 | 70 | 172 | High | 0 |
| 145 | F | 61 | 70 | 163 | High | 0 |
| B128 | M | 81 | 50 | 165 | High | 0 |
| B13 | M | 60 | 71 | 170 | High | 0 |
| B33 | M | 56 | 70 | 171 | High | 0 |
| B5 | M | 84 | 75 | 180 | High | 0 |
| B62 | M | 50 | 83 | 180 | High | 0 |
| B65 | M | 76 | 49 | 166 | High | 0 |
| B72 | F | 81 | 39.6 | 155 | High | 0 |
| B75 | F | 72 | 45 | 158 | High | 0 |
| B8 | M | 60 | 72 | 175 | High | 0 |
| B80 | M | 58 | 81 | 178 | High | 0 |
| B96 | M | 52 | 85 | 175 | High | 0 |
| 89 | M | 81 | 75 | 168 | High | 0 |
| B53 | M | 59 | 78.5 | 170 | High | 0 |
| B148 | M | 58 | 48 | 170 | High | 0 |
| B157 | M | 71 | 56.5 | 168 | High | 0 |
| B169 | M | 91 | 83 | 178 | High | 0 |
| B171 | F | 63 | 55 | 161 | High | 0 |
| B185 | M | 70 | 89 | 170 | High | 0 |
| B192 | M | 74 | 93 | 180 | High | 0 |
| B153 | M | 70 | 86 | 179 | High | 0 |
| B179 | M | 70 | 83 | 178 | High | 0 |

1. NMIBC with hematuria

| Num | Sex | Age | Weight(Kg) | Height(cm) | Grade | Red cell（cells/μL） |
| --- | --- | --- | --- | --- | --- | --- |
| B15 | F | 44 | 57 | 160 | Low | 200 |
| B24 | M | 75 | 51.5 | 170 | Low | 200 |
| B67 | M | 60 | 79 | 170 | Low | 80 |
| B94 | M | 76 | 65 | 168 | Low | 200 |
| 121 | F | 62 | 52 | 154 | Low | 25 |
| 133 | F | 70 | 70 | 164 | Low | 25 |
| 143 | M | 48 | 78 | 180 | Low | 25 |
| 1 | M | 60 | 62 | 170 | Low | 25 |
| 13 | M | 69 | 71 | 172 | Low | 200 |
| 19 | M | 64 | 88 | 182 | Low | 200 |
| 25 | M | 75 | 76 | 179 | Low | 200 |
| 30 | F | 60 | 56 | 164 | Low | 25 |
| 84 | M | 67 | 88.5 | 175 | Low | 200 |
| B156 | M | 71 | 55 | 160 | Low | 25 |
| B160 | F | 68 | 57 | 160 | Low | 25 |
| B184 | M | 89 | 52 | 174 | Low | 200 |
| B69 | M | 66 | 70 | 172 | Low | 200 |
| B26 | F | 53 | 70 | 168 | Low | 200 |
| B2 | F | 51 | 56 | 161 | High | 200 |
| B3 | M | 90 | 61 | 170 | High | 200 |
| B11 | M | 82 | 65 | 170 | High | 80 |
| B21 | M | 62 | 80 | 178 | High | 25 |
| B34 | M | 62 | 78 | 170 | High | 200 |
| B37 | M | 62 | 65 | 170 | High | 200 |
| B39 | M | 66 | 68 | 166 | High | 200 |
| B56 | F | 69 | 60 | 162 | High | 200 |
| B63 | M | 64 | 78 | 170 | High | 200 |
| B66 | M | 62 | 80 | 172 | High | 80 |
| B71 | M | 88 | 46 | 159 | High | 200 |
| B73 | M | 62 | 56 | 161 | High | 200 |
| B74 | M | 67 | 54 | 167 | High | 25 |
| B82 | M | 65 | 66 | 170 | High | 200 |
| B92 | M | 68 | 55 | 173 | High | 25 |
| B93 | M | 83 | 67 | 179 | High | 200 |
| B100 | F | 78 | 44 | 150 | High | 200 |
| B120 | M | 80 | 80 | 171 | High | 200 |
| BLG | M | 66 | 69 | 165 | High | 80 |
| B130 | M | 82 | 78 | 175 | High | 200 |
| B137 | F | 68 | 62.5 | 156 | High | 200 |
| 93 | M | 68 | 71 | 167 | High | 200 |
| 95 | F | 60 | 70 | 163 | High | 25 |
| 97 | M | 76 | 65 | 172 | High | 200 |
| 98 | M | 82 | 62 | 162 | High | 80 |
| 108 | F | 48 | 70 | 168 | High | 200 |
| 112 | M | 50 | 80 | 178 | High | 25 |
| 114 | M | 54 | 74 | 174 | High | 200 |
| 115 | M | 60 | 80 | 170 | High | 25 |
| 123 | M | 83 | 74 | 173 | High | 200 |
| 127 | F | 67 | 63 | 160 | High | 200 |
| 135 | M | 59 | 62 | 157 | High | 200 |
| 144 | M | 71 | 72 | 168 | High | 200 |
| 8 | F | 88 | 69 | 160 | High | 200 |
| 15 | M | 89 | 61 | 170 | High | 200 |
| 17 | M | 81 | 73 | 170 | High | 200 |
| 20 | F | 84 | 55 | 150 | High | 200 |
| 24 | M | 75 | 60 | 170 | High | 25 |
| 31 | M | 76 | 80 | 168 | High | 80 |
| 37 | M | 62 | 80 | 170 | High | 80 |
| 45 | M | 60 | 54 | 165 | High | 200 |
| 46 | M | 67 | 50 | 155 | High | 200 |
| 53 | M | 75 | 75 | 183 | High | 200 |
| 54 | F | 76 | 51 | 151 | High | 200 |
| 64 | M | 69 | 91 | 170 | High | 25 |
| 66 | M | 81 | 64 | 170 | High | 25 |
| 72 | F | 72 | 62 | 165 | High | 25 |
| 83 | M | 58 | 73.5 | 170 | High | 200 |
| B139 | M | 67 | 59.5 | 171 | High | 80 |
| B144 | M | 68 | 56 | 165 | High | 80 |
| B146 | M | 75 | 93 | 180 | High | 200 |
| B150 | M | 78 | 49 | 171 | High | 200 |
| B154 | F | 79 | 39.5 | 158 | High | 200 |
| B161 | M | 64 | 74 | 168 | High | 200 |
| B162 | M | 49 | 96 | 168 | High | 200 |
| B163 | M | 79 | 62.5 | 163 | High | 200 |
| B168 | M | 55 | 72 | 165 | High | 200 |
| B187 | M | 78 | 50 | 171 | High | 200 |
| B189 | M | 69 | 82 | 162 | High | 200 |
| B191 | M | 63 | 91 | 175 | High | 80 |
| B149 | M | 67 | 80 | 172 | High | 200 |
| B159 | M | 55 | 65 | 165 | High | 80 |
| B182 | M | 80 | 55 | 164 | High | 200 |
| B48 | M | 60 | 65 | 172 | High | 80 |
| B51 | F | 68 | 70 | 165 | High | 200 |
| B52 | F | 73 | 60 | 173 | High | 80 |
| B109 | M | 54 | 72 | 172 | High | 25 |
| 100 | M | 44 | 60 | 175 | High | 200 |
| B145 | M | 62 | 56 | 165 | High | 80 |

**Table S2** Differential metabolites between NMIBC and control group

| **Variables** | **Metabolites ID** | **Description** | **Score** | **Fold Change (NMIBC/Control)** | ***p*-value** |
| --- | --- | --- | --- | --- | --- |
| 3.26_216.0080n | HMDB04148 | Dopamine 4-sulfate | 46.8 | 1.648644 | 6.25E-05 |
| 4.94_253.0916m/z | HMDB28755 | Aspartyl-Histidine | 44.2 | 0.415178 | 2.77E-08 |
| 5.37_474.1704m/z | HMDB14728 | Doxazosin | 45.3 | 1.814643 | 8.84E-06 |
| 5.40_395.0919m/z | HMDB41723 | Dihydroferulic acid 4-O-glucuronide | 45.4 | 1.580779 | 4.54E-03 |
| 5.49_330.1462m/z | HMDB29111 | Tyrosyl-Methionine | 43.4 | 0.468075 | 3.03E-05 |
| 5.58_254.1251n | HMDB32775 | 3-Hydroxy-carbofuran | 44.2 | 0.627766 | 7.02E-04 |
| 5.78_159.0676n | HMDB12490 | 1,2-Dehydrosalsolinol | 43.7 | 0.167915 | 4.66E-04 |
| 5.91_381.1737m/z | HMDB15613 | Ecabet | 40.1 | 0.471804 | 2.36E-05 |
| 6.09_362.1493m/z | HMDB15296 | Ofloxacin | 47.5 | 6.315622 | 2.15E-02 |
| 6.86_352.2210m/z | HMDB62691 | 15-dehydro-prostaglandin E1(1-) | 40.8 | 6.495557 | 1.15E-04 |
| 6.95_135.0797m/z | HMDB39956 | 4-(Ethoxymethyl)phenol | 44.6 | 4.861999 | 6.67E-13 |
| 7.04_314.1943m/z | HMDB38120 | Ovalicin | 41.9 | 4.575252 | 4.41E-09 |
| 7.42_452.2460m/z | HMDB14326 | Flunisolide | 40.4 | 2.30399 | 1.18E-06 |
| 7.53_292.1558m/z | HMDB39660 | Gossyvertin | 41.4 | 0.556495 | 2.44E-11 |
| 7.69_349.2351m/z | HMDB31049 | Avocadyne 4-acetate | 41.1 | 0.59821 | 4.02E-06 |
| 7.72_360.2722m/z | HMDB40901 | 13-Hydroxy-9-methoxy-10-oxo-11-octadecenoic acid | 44.3 | 0.656021 | 1.37E-04 |
| 8.43_277.2146m/z | HMDB35877 | (Z)-13-Hexadecenoic acid | 42.8 | 1.879568 | 1.87E-04 |
| 8.43_488.2591n | HMDB38732 | alpha-Ionol O-[arabinosyl-(1->6)-glucoside] | 43.5 | 2.180414 | 6.19E-06 |
| 8.71_368.2774m/z | HMDB11541 | MG(0:0/18:4(6Z,9Z,12Z,15Z)/0:0) | 42 | 0.334335 | 3.68E-07 |
| 8.72_319.2032m/z | HMDB15316 | Fluoxymesterone | 40.6 | 0.569809 | 9.57E-08 |
| 8.72_344.2775m/z | HMDB31006 | 1-Acetoxy-2-hydroxy-16-heptadecen-4-one | 42.9 | 0.400807 | 1.02E-03 |

**Table S3** Differential metabolites for NMIBC and control group distinction

| **Metabolites** | **AUC** | ***p*-value** | **Log2 FC** |
| --- | --- | --- | --- |
| 4-Ethoxymethylphenol | 0.84906 | 6.67E-13 | 2.28155 |
| Gossyvertin | 0.82696 | 2.44E-11 | -0.84556 |
| Fluoxymesterone | 0.79965 | 9.57E-08 | -0.81145 |
| Aspartyl-Histidine | 0.77532 | 2.77E-08 | -1.2682 |
| Ovalicin | 0.76539 | 4.41E-09 | 2.193851 |
| Flunisolide | 0.75695 | 1.18E-06 | 1.204134 |
| MG00/1846Z,9Z,12Z,15Z/00 | 0.74926 | 3.68E-07 | -1.58063 |
| Avocadyne 4-acetate | 0.74106 | 4.02E-06 | -0.74128 |
| alpha-Ionol O-arabinosyl-1-6-glucoside | 0.73808 | 6.19E-06 | 1.124602 |
| Doxazosin | 0.73039 | 8.84E-06 | 0.859686 |
| Tyrosyl-Methionine | 0.71512 | 3.03E-05 | -1.09519 |
| 1,2-Dehydrosalsolinol | 0.71003 | 4.66E-04 | -2.5742 |
| Dopamine 4-sulfate | 0.70879 | 6.25E-05 | 0.72128 |
| Ecabet | 0.70804 | 2.36E-05 | -1.08374 |
| Z-13-Hexadecenoic acid | 0.70035 | 1.87E-04 | 0.910401 |
| 13-Hydroxy-9-methoxy-10-oxo-11-octadecenoic acid | 0.69364 | 1.37E-04 | -0.60819 |
| 15-dehydro-prostaglandin E11- | 0.68198 | 1.15E-04 | 2.699453 |
| Dihydroferulic acid 4-O-glucuronide | 0.6641 | 4.54E-03 | 0.660636 |
| 3-Hydroxy-carbofuran | 0.66311 | 7.02E-04 | -0.6717 |
| 1-Acetoxy-2-hydroxy-16-heptadecen-4-one | 0.65566 | 1.02E-03 | -1.31902 |
| Ofloxacin | 0.64635 | 2.15E-02 | 2.658925 |

The metabolites in red were selected to consist panel.

**Table S4** Differential metabolites between low-grade NMIBC and control group

| **Variables** | **Compound ID** | **Description** | **Score** | **Fold Change（Low/Control）** | ***p*-value** |
| --- | --- | --- | --- | --- | --- |
| 3.26_216.0080n | HMDB04148 | Dopamine 4-sulfate | 44.1 | 2.717704 | 4.72E-06 |
| 4.24_181.0591n | HMDB06037 | 8-Hydroxy-7-methylguanine | 52.5 | 1.749907 | 6.47E-05 |
| 4.83_299.1214n | HMDB34681 | 3-Hydroxy-4-butanolide | 41.6 | 3.221117 | 5.11E-05 |
| 4.83_327.0667m/z | HMDB39287 | Theogallin | 40.1 | 2.065018 | 6.01E-05 |
| 5.15_314.1581m/z | HMDB36156 | Deoxynivalenol | 47.4 | 0.540652 | 0.00586 |
| 5.24_237.0622n | HMDB38942 | Methyl 2,3-dihydro-3,5-dihydroxy-2-oxo-3-indoleacetic acid | 39.9 | 1.547514 | 0.001667 |
| 5.27_213.1220m/z | HMDB29128 | Valyl-Hydroxyproline | 47.1 | 0.603284 | 0.001202 |
| 5.37_474.1704m/z | HMDB00972 | 10-Formyltetrahydrofolate | 42.5 | 1.733066 | 0.002138 |
| 5.58_254.1251n | HMDB13067 | Salsoline-1-carboxylate | 42.3 | 0.425771 | 0.000779 |
| 5.72_346.2204m/z | HMDB61062 | 7-hydroxygranisetron | 46.1 | 1.818168 | 1.91E-05 |
| 5.85_143.0727n | HMDB40048 | 3,4-Dihydro-4-[(5-methyl-2-furanyl)methylene]-2H-pyrrole | 42.1 | 1.586776 | 0.000883 |
| 5.85_363.2238n | HMDB34579 | 8-O-Methyloblongine | 44.6 | 1.625597 | 0.000261 |
| 6.25_222.1111m/z | HMDB33303 | Anofinic acid | 40.8 | 1.667312 | 0.000788 |
| 6.32_225.1102m/z | HMDB59719 | Heptylmalonic acid | 48.2 | 1.90035 | 4.94E-06 |
| 6.38_260.1358n | HMDB38256 | Geranyl acetoacetate | 41 | 4.083207 | 5.71E-09 |
| 6.39_282.1321m/z | HMDB29368 | Saxitoxin | 42.6 | 3.35064 | 0.000516 |
| 6.45_107.0848m/z | HMDB59851 | o-Xylene | 43.8 | 2.085905 | 3.15E-06 |
| 6.45_350.0850m/z | HMDB13118 | Xanthurenate-8-O-beta-D-glucoside | 50.7 | 0.474966 | 0.00035 |
| 6.46_366.1738m/z | HMDB34593 | (S)-Codamine | 37.3 | 0.639325 | 0.002491 |
| 6.64_463.1500n | HMDB36630 | Trifolirhizin | 51 | 0.381542 | 6.38E-05 |
| 6.72_332.2411m/z | HMDB61636 | 3-hydroxydecanoyl carnitine | 44.2 | 0.587161 | 3.64E-05 |
| 6.82_328.2100m/z | HMDB40980 | Valdiate | 46.7 | 1.774756 | 4.27E-05 |
| 6.90_292.1923m/z | HMDB40926 | 2-Butyl-5-[2-(4-hydroxy-3-methoxyphenyl)ethyl]furan | 40.1 | 0.46705 | 1.5E-05 |
| 7.15_295.2014m/z | HMDB14511 | Levonorgestrel | 47 | 0.512447 | 0.000299 |
| 7.21_330.1658n | HMDB39056 | (4S,6R)-p-Mentha-1,8-diene-6,7-diol 7-glucoside | 44.2 | 0.419566 | 2.05E-05 |
| 7.25_349.2351m/z | HMDB13221 | Beta-Cortolone | 48.5 | 0.602289 | 2.96E-06 |
| 7.32_318.2076m/z | HMDB60088 | 4-Methoxyestrone | 45.9 | 0.604015 | 8.44E-05 |
| 7.33_246.1505m/z | HMDB59773 | S-3-oxodecanoyl cysteamine | 45.2 | 0.598296 | 6.2E-05 |
| 7.36_240.1710n | HMDB00638 | Dodecanoic acid | 44.9 | 1.588545 | 0.005129 |
| 7.42_452.2460m/z | HMDB14326 | Flunisolide | 43.9 | 2.195991 | 0.000101 |
| 7.53_292.1558m/z | HMDB01944 | Chlorpheniramine | 39.2 | 0.471886 | 4.78E-11 |
| 7.72_360.2722m/z | HMDB40901 | 13-Hydroxy-9-methoxy-10-oxo-11-octadecenoic acid | 41.7 | 0.564161 | 0.000611 |
| 7.93_296.1840m/z | HMDB14333 | Esmolol | 38.6 | 3.358705 | 0.003845 |
| 7.98_342.1711m/z | HMDB32671 | (E)-2',4,4'-Trihydroxy-3-prenylchalcone | 52.6 | 0.634627 | 2.28E-05 |
| 8.02_284.2201m/z | HMDB60055 | Tetranor 12-HETE | 44 | 0.636191 | 5.75E-06 |
| 8.06_229.1243m/z | HMDB37386 | (S)-Menthone 8-thioacetate | 38.8 | 0.541571 | 2.41E-08 |
| 8.06_256.1481n | HMDB15114 | Cyclopentolate | 40.4 | 0.574832 | 4.38E-09 |
| 8.13_268.2022n | HMDB00806 | Myristic acid | 46.1 | 2.176422 | 0.001275 |
| 8.19_316.2464m/z | HMDB62631 | O-decanoyl-L-carnitine | 48.7 | 0.57569 | 0.001003 |
| 8.30_488.2591n | HMDB38732 | alpha-Ionol O-[arabinosyl-(1->6)-glucoside] | 41.9 | 2.612201 | 0.000185 |
| 8.33_320.1869m/z | HMDB00530 | 6-Ketoestriol | 42.2 | 0.530827 | 1.09E-07 |
| 8.43_372.2182m/z | HMDB31877 | Acetylsalvipisone | 39.5 | 0.48755 | 0.000241 |
| 8.44_344.1870m/z | HMDB30270 | Anomurine | 40.3 | 0.629404 | 1.4E-05 |
| 8.50_384.2723m/z | HMDB13332 | 3-Hydroxy-5, 8-tetradecadiencarnitine | 41.6 | 0.479317 | 0.000191 |
| 8.51_330.2620m/z | HMDB13321 | Undecanoylcarnitine | 48.6 | 0.438325 | 0.000682 |
| 8.57_571.3191m/z | HMDB60847 | N-Monodesmethyl-rizatriptan | 40.7 | 0.559151 | 0.002173 |
| 8.62_334.1663m/z | HMDB14808 | Pirenzepine | 42.6 | 0.536324 | 3.54E-06 |
| 8.71_368.2774m/z | HMDB00268 | Tetrahydrocorticosterone | 41.7 | 0.261641 | 5E-07 |
| 8.74_513.2640m/z | HMDB60120 | 10-Hydroxy-octadec-12Z-enoate-9-beta-D-glucuronide | 44.2 | 2.058325 | 0.000299 |
| 8.76_386.2879m/z | HMDB13330 | 3-Hydroxy-cis-5-tetradecenoylcarnitine | 42.6 | 0.379984 | 4.2E-09 |
| 8.85_410.2878m/z | HMDB61640 | 3-hydroxytetradecanoyl carnitine | 40.4 | 0.439258 | 1.55E-05 |

**Table S5** Differential metabolites for low grade NMIBC distinction

| **Metabolites** | **AUC** | ***p*-value** | **Log2 FC** |
| --- | --- | --- | --- |
| Chlorpheniramine | 0.88776 | 4.78E-11 | -1.08349 |
| Cyclopentolate | 0.86395 | 4.38E-09 | -0.79879 |
| 3-Hydroxy-cis-5-tetradecenoylcarnitine | 0.85147 | 4.2E-09 | -1.39599 |
| S-Menthone 8-thioacetate | 0.84354 | 2.41E-08 | -0.88478 |
| Geranyl acetoacetate | 0.8339 | 5.71E-09 | 2.029703 |
| 6-Ketoestriol | 0.8237 | 1.09E-07 | -0.91369 |
| Tetranor 12-HETE | 0.80102 | 5.75E-06 | -0.65247 |
| Beta-Cortolone | 0.79762 | 2.96E-06 | -0.73147 |
| Tetrahydrocorticosterone | 0.79649 | 5E-07 | -1.93434 |
| Dopamine 4-sulfate | 0.78741 | 4.72E-06 | 1.442388 |
| o-Xylene | 0.78515 | 3.15E-06 | 1.060673 |
| Heptylmalonic acid | 0.78345 | 4.94E-06 | 0.926265 |
| Pirenzepine | 0.78118 | 3.54E-06 | -0.89882 |
| Anomurine | 0.77551 | 1.4E-05 | -0.66794 |
| Theogallin | 0.76587 | 6.01E-05 | 1.046154 |
| Levonorgestrel | 0.76474 | 0.000299 | -0.96452 |
| 2-Butyl-5-2-4-hydroxy-3-methoxyphenylethylfuran | 0.76417 | 1.5E-05 | -1.09835 |
| S-3-oxodecanoyl cysteamine | 0.76417 | 6.2E-05 | -0.74107 |
| E-2',4,4'-Trihydroxy-3-prenylchalcone | 0.76247 | 2.28E-05 | -0.65602 |
| 3-hydroxytetradecanoyl carnitine | 0.75794 | 1.55E-05 | -1.18686 |
| 7-hydroxygranisetron | 0.75794 | 1.91E-05 | 0.862486 |
| 4S,6R-p-Mentha-1,8-diene-6,7-diol 7-glucoside | 0.7568 | 2.05E-05 | -1.25303 |
| Deoxynivalenol | 0.75454 | 0.00586 | -0.88723 |
| Trifolirhizin | 0.74546 | 6.38E-05 | -1.39009 |
| 3-Hydroxy-5, 8-tetradecadiencarnitine | 0.74093 | 0.000191 | -1.06095 |
| Valdiate | 0.7398 | 4.27E-05 | 0.827621 |
| Flunisolide | 0.73923 | 0.000101 | 1.134872 |
| Xanthurenate-8-O-beta-D-glucoside | 0.73753 | 0.00035 | -1.0741 |
| 3-Hydroxy-4-butanolide | 0.73639 | 5.11E-05 | 1.687561 |
| alpha-Ionol O-arabinosyl-1-6-glucoside | 0.73639 | 0.000185 | 1.385266 |
| 10-Hydroxy-octadec-12Z-enoate-9-beta-D-glucuronide | 0.73583 | 0.000299 | 1.041471 |
| 4-Methoxyestrone | 0.73583 | 8.44E-05 | -0.72734 |
| 3-hydroxydecanoyl carnitine | 0.73073 | 3.64E-05 | -0.76817 |
| 8-O-Methyloblongine | 0.73016 | 0.000261 | 0.70097 |
| Anofinic acid | 0.72902 | 0.000788 | 0.737524 |
| 8-Hydroxy-7-methylguanine | 0.72732 | 6.47E-05 | 0.807278 |
| Acetylsalvipisone | 0.72392 | 0.000241 | -1.03638 |
| Salsoline-1-carboxylate | 0.71542 | 0.000779 | -1.23185 |
| 3,4-Dihydro-4-5-methyl-2-furanylmethylene-2H-pyrrole | 0.71429 | 0.000883 | 0.666099 |
| Esmolol | 0.71372 | 0.003845 | 1.747905 |
| Myristic acid | 0.71088 | 0.001275 | 1.121958 |
| Valyl-Hydroxyproline | 0.70862 | 0.001202 | -0.72909 |
| 10-Formyltetrahydrofolate | 0.70748 | 0.002138 | 0.793327 |
| N-Monodesmethyl-rizatriptan | 0.70692 | 0.002173 | -0.83869 |
| O-decanoyl-L-carnitine | 0.70578 | 0.001003 | -0.79664 |
| S-Codamine | 0.70181 | 0.002491 | -0.64538 |
| Saxitoxin | 0.7004 | 0.000516 | 1.744437 |
| 13-Hydroxy-9-methoxy-10-oxo-11-octadecenoic acid | 0.69955 | 0.000611 | -0.82582 |
| Undecanoylcarnitine | 0.69558 | 0.000682 | -1.18993 |
| Methyl 2,3-dihydro-3,5-dihydroxy-2-oxo-3-indoleacetic acid | 0.68594 | 0.001667 | 0.629952 |
| Dodecanoic acid | 0.68367 | 0.005129 | 0.667706 |

The metabolites in red were selected to consist panel.

**Table S6** Differential metabolites between high and low grade NMIBC without hematuria

| **Variables** | **Compound ID** | **Description** | **Score** | **Fold Change (High/Low)** | ***p*-value** |
| --- | --- | --- | --- | --- | --- |
| 4.42_314.1561n | HMDB41513 | Isopropyl apiosylglucoside | 43.2 | 0.454003 | 0.029295 |
| 4.46_316.0776n | HMDB00796 | N-Acetyl-4-O-acetylneuraminic acid | 45.9 | 0.624966 | 0.003652 |
| 4.88_240.1005m/z | HMDB14643 | Tolmetin | 40.6 | 3.438438 | 0.027762 |
| 5.17_346.2205m/z | HMDB61062 | 7-hydroxygranisetron | 42.7 | 0.507714 | 0.038771 |
| 5.41_156.1010m/z | HMDB40174 | 4-Ethyl-1,2-benzenediol | 45 | 1.894402 | 0.046978 |
| 5.47_251.1375m/z | HMDB29876 | Paucine | 42.4 | 1.857081 | 0.000548 |
| 5.94_208.0592m/z | HMDB00978 | 4-(2-Aminophenyl)-2,4-dioxobutanoic acid | 40.2 | 3.435115 | 0.003507 |
| 6.84_305.1640n | HMDB60994 | alpha-Hydroxymetoprolol | 44.2 | 1.642462 | 0.035389 |
| 7.25_228.1944m/z | HMDB38258 | Neryl propionate | 43.9 | 0.779837 | 0.026053 |
| 7.35_312.2144m/z | HMDB13202 | 6-Keto-decanoylcarnitine | 53.1 | 0.567669 | 0.031854 |
| 7.55_332.2407m/z | HMDB61636 | 3-hydroxydecanoyl carnitine | 43.8 | 1.694544 | 0.025893 |
| 8.41_466.2539n | HMDB10365 | 3-alpha-hydroxy-5-alpha-androstane-17-one 3-D-glucuronide | 48.5 | 0.704947 | 0.002518 |
| 8.66_342.2619m/z | HMDB13164 | 2-Hydroxylauroylcarnitine | 51.4 | 1.508406 | 0.034631 |
| 8.76_386.2879m/z | HMDB37961 | gamma-Eudesmol rhamnoside | 50.5 | 2.902715 | 0.017958 |
| 8.93_356.2775m/z | HMDB35379 | Sterebin E | 45.7 | 2.081135 | 0.006217 |

**Table S7** Differential metabolites for high and low grade NMIBC without hematuria distinction

| **Metabolites** | **AUC** | ***p*-value** | **Log2 FC** |
| --- | --- | --- | --- |
| Paucine | 0.73037 | 0.000548 | 0.893037 |
| Sterebin E | 0.70077 | 0.006217 | 1.057371 |
| 3-alpha-hydroxy-5-alpha-androstane-17-one 3-D-glucuronide | 0.67117 | 0.002518 | -0.50441 |
| 4-2-Aminophenyl-2,4-dioxobutanoic acid | 0.66988 | 0.003507 | 1.780358 |
| gamma-Eudesmol rhamnoside | 0.66538 | 0.017958 | 1.537403 |
| 6-Keto-decanoylcarnitine | 0.66088 | 0.031854 | -0.81688 |
| N-Acetyl-4-O-acetylneuraminic acid | 0.65766 | 0.003652 | -0.67815 |
| Tolmetin | 0.64736 | 0.027762 | 1.781753 |
| Neryl propionate | 0.64672 | 0.026053 | -0.35876 |
| Isopropyl apiosylglucoside | 0.64414 | 0.029295 | -1.13923 |
| alpha-Hydroxymetoprolol | 0.63707 | 0.035389 | 0.71586 |
| 3-hydroxydecanoyl carnitine | 0.63642 | 0.025893 | 0.760897 |
| 7-hydroxygranisetron | 0.63385 | 0.038771 | -0.97791 |
| 4-Ethyl-1,2-benzenediol | 0.63256 | 0.046978 | 0.921743 |
| 2-Hydroxylauroylcarnitine | 0.63127 | 0.034631 | 0.593024 |

The metabolites in red were selected to consist panel.

**Table S8** Differential metabolites between high and low grade NMIBC with hematuria

| **Variables** | **Compound ID** | **Description** | **Score** | **Fold Change (High/Low)** | ***p*-value** |
| --- | --- | --- | --- | --- | --- |
| 2.21_220.1278m/z | HMDB28688 | Alanyl-Hydroxyproline | 44 | 2.437006 | 0.010091 |
| 2.37_225.0857m/z | HMDB11631 | L-3-Hydroxykynurenine | 48 | 2.399375 | 0.015131 |
| 4.41_193.0961m/z | HMDB04073 | 5-Hydroxyindoleacetaldehyde | 44.5 | 2.337635 | 0.026881 |
| 4.60_191.0572n | HMDB60400 | 5-Phenyl-1,3-oxazinane-2,4-dione | 48.1 | 2.065164 | 0.001239 |
| 5.59_219.1120m/z | HMDB62497 | N-acetyl-5-methoxykynuramine | 35.2 | 105.6031 | 0.007888 |
| 6.24_378.1440m/z | HMDB00988 | S-Adenosylmethioninamine | 42.5 | 1755.201 | 0.005231 |
| 6.35_345.1524m/z | HMDB31694 | Butyl (S)-3-hydroxybutyrate glucoside | 43.6 | 2.583599 | 0.012374 |
| 6.99_275.1295m/z | HMDB28887 | Histidinyl-Histidine | 39.2 | 54.06958 | 0.003623 |
| 7.00_170.0591m/z | HMDB00734 | Indoleacrylic acid | 43.4 | 0.536235 | 0.015182 |
| 7.48_245.0905m/z | HMDB06005 | Indolylacryloylglycine | 45.1 | 0.392121 | 0.001795 |

**Table S9** Differential metabolites for high and low grade NMIBC with hematuria distinction

| **Metabolites** | **AUC** | ***p*-value** | **Log2 FC** |
| --- | --- | --- | --- |
| Indolylacryloylglycine | 0.75443 | 0.001795 | -1.35063 |
| 5-Phenyl-1,3-oxazinane-2,4-dione | 0.74316 | 0.001239 | 1.046256 |
| Histidinyl-Histidine | 0.72866 | 0.003623 | 5.756745 |
| Indoleacrylic acid | 0.71739 | 0.015182 | -0.89906 |
| N-acetyl-5-methoxykynuramine | 0.70934 | 0.007888 | 6.722509 |
| S-Adenosylmethioninamine | 0.69646 | 0.005231 | 10.77742 |
| L-3-Hydroxykynurenine | 0.69243 | 0.015131 | 1.262659 |
| Alanyl-Hydroxyproline | 0.69002 | 0.010091 | 1.28511 |
| Butyl S-3-hydroxybutyrate glucoside | 0.67794 | 0.012374 | 1.369382 |
| 5-Hydroxyindoleacetaldehyde | 0.67432 | 0.026881 | 1.22505 |

The metabolites in red were selected to consist panel.
